# Effects of Physical Activity on Executive Function and Emotional Regulation in Children and Adolescents with Neurodevelopmental Disorders: A Systematic Review and Meta-Analysis

**DOI:** 10.3390/healthcare13192415

**Published:** 2025-09-24

**Authors:** María del Carmen Carcelén-Fraile, Fidel Hita-Contreras, María Aurora Mesas-Aróstegui, Agustín Aibar-Almazán

**Affiliations:** 1 Department of Educational Sciences, Faculty of Social Sciences, University of Atlántico Medio, 35017 Las Palmas de Gran Canaria, Spain; 2 International Scientific Association on Innovation in Education and Health (ACIINES), 23007 Jaén, Spain; 3 International Network of Educational Law, 35017 Las Palmas de Gran Canaria, Spain; 4 Department of Health Sciences, Faculty of Health Sciences, University of Jaén, 23071 Jaén, Spain; 5 Pediatrics Department, Hospital of Guadix, 18500 Granada, Spain; maria.mesas.sspa@juntadeandalucia.es

**Keywords:** neurodevelopmental disorders, physical activity, executive function, emotional regulation, children, adolescents, systematic review, meta-analysis

## Abstract

**Background/Objectives:** Children and adolescents with neurodevelopmental disorders (NDDs) often experience deficits in executive functioning and emotional regulation, which impact their academic, social, and behavioral development. While physical activity is increasingly recognized as a promising non-pharmacological intervention, the specific effects on cognitive and emotional domains remain heterogeneous. This systematic review and meta-analysis aimed to assess the efficacy of physical–motor interventions in improving executive functions and emotional regulation in youths with NDDs. **Methods**: Following PRISMA 2020 guidelines, a comprehensive search of five databases was conducted (2010–2024) to identify randomized controlled trials (RCTs) evaluating the effects of structured physical activity programs on executive and emotional outcomes in children and adolescents diagnosed with NDDs. A total of 22 RCTs were included in the qualitative synthesis, while 16 were included in the quantitative analysis. Effect sizes were calculated using a random effects model, while heterogeneity was assessed with the Q, I^2^, Tau^2^, and Egger’s tests. **Results**: Physical activity interventions demonstrated a non-significant effect on executive functioning (g = 0.492; *p* = 0.215; 95% CI: −0.286 to 1.269). Although the point estimate suggested a small-to-moderate effect, the wide confidence interval and lack of statistical significance prevent firm conclusions. In contrast, a large and significant effect was observed on emotional regulation outcomes (g = −1.204; *p* < 0.001; 95% CI: −1.688 to −0.655), despite moderate heterogeneity (I^2^ = 72.3%). Several studies also reported specific improvements in working memory, cognitive flexibility, and emotional control. **Conclusions**: Structured physical activity may be an effective complementary intervention for improving emotional regulation in youth with NDDs, with less consistent evidence for executive functioning. Future research should clarify optimal protocols and target populations to enhance intervention effectiveness.

## 1. Introduction

Neurodevelopmental disorders (NDD) constitute a heterogeneous group of conditions that emerge during child development and typically manifest in early childhood [1]. Among the most prevalent NDDs are attention-deficit/hyperactivity disorder (ADHD), autism spectrum disorder (ASD), intellectual disability (ID), and developmental coordination disorder (DCD) [2]. These conditions affect approximately one in six children worldwide, and their incidence has increased significantly in recent decades [3].

NDDs are characterized by persistent impairments in key cognitive processes, such as attention, working memory, response inhibition, and cognitive flexibility, which form the core of executive functioning [4]. Difficulties in emotional regulation, mood disorders (such as anxiety and depression), behavioral problems (aggression and impulsivity), and limitations in social and academic adjustment are also common [5,6]. These impairments not only compromise academic performance and social interaction but are also associated with an increased risk of social isolation, low self-esteem, prolonged dependency, and psychiatric comorbidities in adolescence and adulthood [7,8]. At the functional level, these disorders often coexist, share multiple etiological mechanisms (genetic, neurological, and environmental), and present a high variability in symptom expression, which complicates their diagnosis and treatment [9]. Therefore, NDDs require interdisciplinary intervention approaches that not only address the core symptoms but also promote comprehensive cognitive, emotional, and social development.

Among the NDDS, there are ADHD, ASD, and CD. Although they present distinct clinical profiles and specific etiologies, they are all classified as neurodevelopmental disorders and share core alterations in transversal domains such as executive function, emotional regulation, and motor coordination. These common difficulties significantly affect academic performance, social adaptation, and quality of life, and are precisely the targets of physical and motor interventions. Furthermore, the recent literature in relation to developmental neuroscience has highlighted the usefulness of transdiagnostic approaches, which allow for the analysis of shared mechanisms beyond traditional diagnostic categories.

In recent years, there has been a significant increase in interest in non-pharmacological interventions as a complement or alternative to traditional treatments in order to address the cognitive, emotional, and behavioral challenges associated with NDDs [10]. Within these strategies, physical activity (PA) has emerged as a promising option due to its low cost, ease of implementation, minimal adverse effects, and its potential to be integrated into both school and clinical settings [11,12]. Numerous studies have documented that regular PA practice can generate significant improvements in executive functions such as response inhibition, working memory, planning, and cognitive flexibility, all of which are essential for learning and behavioral control [13,14,15]. These benefits have been particularly evident in populations with ADHD and ASD, where executive functions are often compromised. Furthermore, physical exercise has been observed to contribute to improved emotional self-regulation, decreasing levels of anxiety, depression, irritability, and aggression, as well as promoting more adaptive social behaviors [16,17].

From a neurobiological perspective, PA has been shown to activate multiple brain systems involved in cognitive and emotional processing [18]. In particular, it stimulates neurogenesis, synaptogenesis, and the release of neurotransmitters such as dopamine and serotonin, which are essential for motivation, mood, and attention [19]. Furthermore, it has been shown to increase the release of BDNF (brain-derived neurotrophic factor), a protein essential for synaptic plasticity, learning, and memory consolidation [20].

Physical activity-based interventions can take a variety of forms, from structured aerobic exercise programs, motor games, martial arts, therapeutic dance, or team sports to strategies combined with cognitive or sensorimotor training [21]. Those of moderate-to-vigorous intensity, performed regularly (2 to 3 times per week), have shown the most robust effects, both in clinical and school settings [22]. Furthermore, these activities offer a motivating, playful, and socially enriching context, which is especially relevant for children with NDDs, who often have difficulty maintaining attention and regulating their emotions in formal or structured contexts. Although numerous individual studies have reported benefits in specific cognitive and emotional domains, the findings remain heterogeneous due to variability in the intervention types, diagnostic categories, and assessment tools used. Therefore, a systematic and quantitative synthesis is needed to comprehensively understand the impact of physical activity on cognitive and emotional functioning in neurodevelopmental disorders [23].

Based on the above, the objective of this study is to synthesize and quantify the available evidence on the effects of physical activity-based interventions on the executive, emotional, and behavioral functioning of children and adolescents with neurodevelopmental disorders in order to evaluate their overall effectiveness and provide guidance for future research and clinical implications.

## 2. Materials and Methods

### 2.1. Information Sources

A systematic literature search was conducted between October and November 2024 in the following electronic databases: PubMed, Scopus, Web of Science (WOS), and CINAHL. These databases were selected for their relevance and interdisciplinary coverage in medicine, psychology, neuroscience, education, and exercise science.

The search was limited to publications in English or Spanish and studies published between January 2020 and January 2025, with the aim of capturing the most up-to-date scientific evidence on the effects of physical activity on cognitive, emotional, and behavioral functioning in children and adolescents with neurodevelopmental disorders (NDDs), including ADHD, ASD, intellectual disability, and developmental coordination disorder.

In addition, a complementary manual search was conducted in the reference lists of the selected articles, previous systematic reviews, and related meta-analyses to identify potentially relevant studies not retrieved in the electronic search. This review protocol was prospectively registered in the PROSPERO database (registration number: CRD420251108172).

### 2.2. Search Strategy

Different keywords were combined in the following search string: (“physical activity” OR “exercise” OR “motor training” OR “aerobic exercise” OR “movement program”) AND (“neurodevelopmental disorders” OR “ADHD” OR “attention-deficit/hyperactivity disorder” OR “autism” OR “ASD” OR “intellectual disability” OR “developmental coordination disorder”) AND (“executive function” OR “cognitive function” OR “emotional regulation” OR “mental health” OR “behavior” OR “behavior” OR “anxiety” OR “depression”). The strategy was adapted to each of the databases consulted (PubMed, Scopus, Web of Science, and CINAHL), applying filters by year of publication (2020–2024), human participants, age (5–17 years), and language (Spanish or English). In addition, a manual search was performed on the reference lists of included studies and relevant reviews to identify potentially eligible articles not retrieved in the electronic search.

### 2.3. Inclusion Criteria

Selected articles had to meet the following criteria: (i) studies had to be randomized clinical trials (RCTs); (ii) the intervention evaluated had to be based exclusively on structured physical activity (e.g., aerobic exercise, motor, perceptual, or multicomponent training); (iii) participants had to be children or adolescents (aged 5–17 years) with a clinical diagnosis of a neurodevelopmental disorder (ADHD, ASD, intellectual disability, or developmental coordination disorder), established through validated diagnostic tools or professional assessment; and (iv) studies had to report at least one quantitative outcome related to cognitive (executive functions), emotional (emotional regulation, anxiety, or depression), or behavioral (e.g., impulsivity or aggression) variables, assessed using psychometric instruments or validated neuropsychological tasks.

### 2.4. Exclusion Criteria

Articles were excluded if they met any of the following criteria: (i) studies without a control group or a valid comparison group; (ii) studies that did not include outcome measures related to the cognitive, emotional, or behavioral variables of interest; (iii) studies that included participants with other serious clinical conditions not within the neurodevelopmental spectrum, such as epilepsy, neurodegenerative diseases, cancer, musculoskeletal, cardiovascular, or renal disorders; and (iv) studies in which participants did not meet the minimum attendance rate required for the intervention program.

### 2.5. Study Selection Process

The process of selecting studies started with the removal of duplicate records and articles without accessible abstracts. Next, titles and abstracts were thoroughly reviewed to exclude those that did not align with the predetermined eligibility criteria. Articles that advanced past this stage were examined in full-text format in order to assess their appropriateness for inclusion in the systematic review and meta-analysis. To maintain objectivity and minimize potential bias, two authors (F.H.-C. and A.A.-A.) independently carried out the selection process. In the case of disagreement regarding a study’s eligibility, a third author (M.d.C.C.-F.) was consulted to provide a final decision and reach a consensus. This rigorous approach ensured that all included studies were relevant and conformed to the defined criteria.

### 2.6. Data Extraction

The main outcome evaluated in this study was the cognitive, emotional, and behavioral performance of children and adolescents with neurodevelopmental disorders who engaged in physical activity-based interventions. Information extracted from the studies included details such as the authors, publication year, country of origin, participant characteristics (sample size, average age, specific NDD diagnosis, and group distribution), research design, variables measured, tools used for assessment, a comprehensive description of the intervention (including its type, frequency, duration, and intensity), assessment time points (e.g., pretest, posttest, and follow-up, when available), dropout rates, any reported adverse events, and key statistical results related to the outcomes of interest.

### 2.7. Assessment of Methodological Quality

The methodological quality of the studies included in the review was evaluated using the PEDro scale, a tool specifically developed to assess randomized controlled trials [24]. This scale comprises 11 items; however, the first item (“eligibility criteria”) serves solely for descriptive purposes and is not factored into the final score, making the highest possible score 10 points. Each criterion is scored as “Yes” (1 point) if met or “No” (0 points) if not. The quality of the studies was categorized based on the following scoring ranges: 0–3 points indicated “low quality,” 4–5 points indicated “acceptable quality,” 6–8 points indicated “good quality,” and scores of 9 or above were considered “excellent quality” [25].

### 2.8. Analytical Decisions for Meta-Analysis

The meta-analysis findings were presented using forest plots, which included essential details such as the lead author, publication year, sample size, and individual effect sizes calculated using Hedges’ g. Each effect size was accompanied by its 95% confidence interval and p-value. To evaluate the reliability of the results, a sensitivity analysis was conducted by removing studies with duplicate data, outliers, or single-case reports that could potentially skew the overall results. These findings were then compared to the full meta-analysis to determine the consistency and robustness of the outcomes. For the subgroup analysis, studies were divided into two main categories based on the outcome variables: (1) mental health, encompassing emotional and behavioral indicators (e.g., anxiety, depression, emotional regulation, and aggression) and (2) cognitive functioning, focusing on executive function measures such as working memory, inhibitory control, and cognitive flexibility. Separate meta-analyses were performed for each subgroup to better understand the variation in effects and to clarify the specific influence of physical activity on each domain. To manage variability among the included studies, a random effects model was used, accounting for true differences in effect sizes across studies and enabling a broader generalization of the results. Statistical heterogeneity was evaluated using Cochran’s Q test and the I^2^ statistic, with I^2^ values over 50% indicating moderate-to-high heterogeneity, suggesting possible methodological, contextual, or population-based differences. Publication bias was assessed through funnel plot analysis, helping to identify the potential distortions caused by the preferential reporting of significant or favorable results. A visual inspection of the funnel plot was supplemented with statistical tools such as Egger’s test to detect asymmetries that might indicate the presence of bias in the body of literature reviewed.

## 3. Results

### 3.1. Study Selection Process

**Figure 1:**
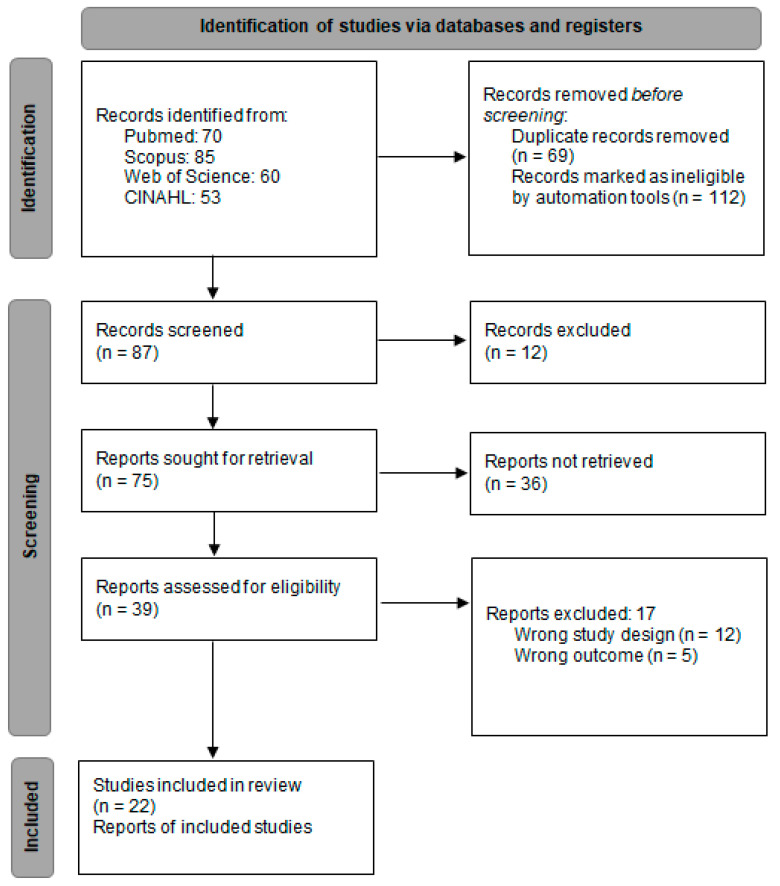
Study selection process flow chart.

A total of 268 records were initially identified through searches across the selected databases. This number was reduced by applying filters for study type (original articles and randomized controlled trials), publication language (English and Spanish), and target population (children and adolescents). Additional refinement was achieved through a title and abstract keyword search and the elimination of duplicates, resulting in 87 unique articles. During the first screening stage, titles and abstracts were reviewed, and 42 articles were shortlisted for full-text evaluation based on their potential relevance. After a comprehensive assessment of these texts, 22 studies fulfilled all the inclusion criteria and were incorporated into the qualitative synthesis and meta-analysis. The remaining 17 studies were excluded due to factors such as inappropriate study design, irrelevant outcome measures, or the absence of interventions centered on physical activity. The entire selection procedure is outlined in Figure 1, in accordance with the PRISMA 2020 framework.

### 3.2. Methodological Quality

The methodological quality of the 22 studies included in this systematic review was assessed using the PEDro scale, which evaluates key aspects of experimental design in randomized controlled clinical trials. This scale is scored from 0 to 10, excluding the item on eligibility criteria, which is not counted in the total. Scores ranged from 4/10 to 7/10, indicating moderate methodological quality in most studies. No study achieved the maximum score or was classified as low quality (≤3 points), although only one achieved a score considered high (≥7 points). Overall, 59% of the studies scored between 5 and 6 points, and 32% only scored 4 points. These findings indicate that although the included trials present a methodologically sound basic structure (randomization, intergroup analysis, complete data), they lack key procedures that reduce the risk of bias and improve internal validity, such as blinding and control for bias in the analysis. Therefore, caution should be exercised in interpreting the results, and the need to improve methodological design in future research on physical interventions in populations with neurodevelopmental disorders is emphasized. A detailed assessment of the methodological quality is provided in Table 1.

### 3.3. Characteristics of the Studies

All studies included in this systematic review and meta-analysis were randomized clinical trials (RCTs), published between 2019 and 2023, and conducted in a wide variety of countries. These include China [26,28,30], Brazil [27], Switzerland [29], Iran [32,37,40,41], Italy [35], South Korea [34,38], Serbia [42], Tunisia [39], and others. In total, 22 studies evaluating the effects of physical therapy interventions on 1,016 children and adolescents with different neurodevelopmental disorders (NDDs), mainly ADHD, autism spectrum disorder (ASD), intellectual disability (ID), developmental coordination disorder (DCD), and learning disorders, are included. The interventions consisted of structured physical activity programs, including judo; soccer; swimming; dance; taekwondo; karate; Sports, Play, and Active Recreation for Kids (SPARK); active video games (exergaming); circuit training; motor exercises; and combined activities. These were implemented primarily in school or clinical settings, with a general duration of 4 to 36 weeks. Sessions ranged from 30 to 70 min, with a frequency of 2 to 3 times per week, reaching up to 144 sessions in some cases.

In total, 550 participants were part of the intervention groups, while 466 were part of the control groups, most of whom received standard care, traditional classes, or no additional intervention. The gender distribution reflected a male predominance, which was consistent with the higher prevalence of NDDs in children. Participants’ ages ranged from 6 to 17 years, with a mean of approximately 10.9 years.

The interventions analyzed in this review showed notable variability in terms of the weekly frequency and total duration of the physical activity programs implemented. Most studies opted for schedules of two or three sessions per week, with the latter being the most prevalent format. Specifically, 17 studies implemented three sessions per week, as was the case with Chang et al. [26], Liang et al. [28], Ji et al. [30], Damanpak and Sabzi [32], Hashem et al. [37], and Zhang et al. [44], thus following international recommendations on physical activity for children with NDDs. On the other hand, nine studies opted for a frequency of twice per week, such as Da Silva et al. [27], Ludyga et al. [29], Mero Piedra et al. [31], and Greco and De Ronzi [36]. Only one study [47] implemented an acute intervention, with a single 30 min exercise session. The total duration ranged from 10 to 144 sessions, with per-session times varying between 30 and 70 min. Studies such as those by Kadri et al. [39] achieved extended programs (approximately 144 sessions), while others, such as Aithal et al. [45], implemented short programs of only 10 sessions. This range reflects both methodological diversity and logistical limitations in educational and clinical settings.

All included studies used randomized clinical trial designs, with a predominant distribution of two parallel groups—intervention and control. However, some studies adopted more complex approaches. For example, Liang et al. [28] and Ji et al. [38] reported designs with more than two groups, allowing for comparisons of multiple experimental modalities or conditions. This methodological variety allows for more detailed analyses, distinguishing between cognitive, behavioral, and emotional effects, as well as justifying the application of subgroup analyses in subsequent statistical steps (Table 2).

### 3.4. Study Results

Of the 22 studies included in this systematic review, all were considered in the qualitative analysis. Although not all assessed cognition globally, most focused on specific executive functions such as sustained attention, inhibitory control, planning, cognitive flexibility, or working memory. Several studies also analyzed emotional and social dimensions, such as anxiety, social skills, or behavioral problems.

Several trials used standardized instruments to measure executive performance. For example, Chang et al. [26] employed the WCST and the Stroop Test, demonstrating improvements in cognitive flexibility and inhibitory control (*p* = 0.002 and *p* = 0.017). Liang et al. [28] reported significant benefits in working memory and executive attention (*p* < 0.01) with tasks such as the Flanker and Trail Making Test. Similarly, Ji et al. [30] obtained marked improvements in inhibition and memory (*p* < 0.001) after a soccer intervention. Other studies with positive results include Da Silva et al. [27] (cognitive flexibility, *p* = 0.042), Ryu et al. [34] (emotional control and inhibition, *p* < 0.05), and Ludyga et al. [29] (working memory, *p* = 0.030). However, some studies did not find statistically significant differences. For example, Mero Piedra et al. [31] observed no improvements in attention or inhibition in children with intellectual disabilities (*p* > 0.05), despite implementing a structured physical education intervention.

In the emotional and social sphere, positive results were highlighted in studies such as Wang et al. [33] (reduction in emotional and behavioral problems, *p* < 0.001), Perić et al. [42] (decrease in aggression, anxiety, and depression, *p* < 0.05), and Sabzi et al. [43] (improvements in behavior and anxiety, *p* = 0.003 and *p* = 0.017). Aithal et al. [45] also showed a favorable effect on emotional and social well-being through dance therapy (*p* = 0.02). Similarly, Sani et al. [41] found significant improvements in sustained attention after perceptual–motor exercises (*p* < 0.05).

Some studies explored technological interventions such as exergaming or active video games. Milajerdi et al. [40] and Ji et al. [38] reported improvements in cognitive flexibility, attention, and inhibition using platforms such as SPARK and stationary bikes with video games. Hashemi et al. [37] also demonstrated improvements in attention, memory, and planning with Nintendo Wii Fit.

### 3.5. Meta-Analysis

**Figure 2:**
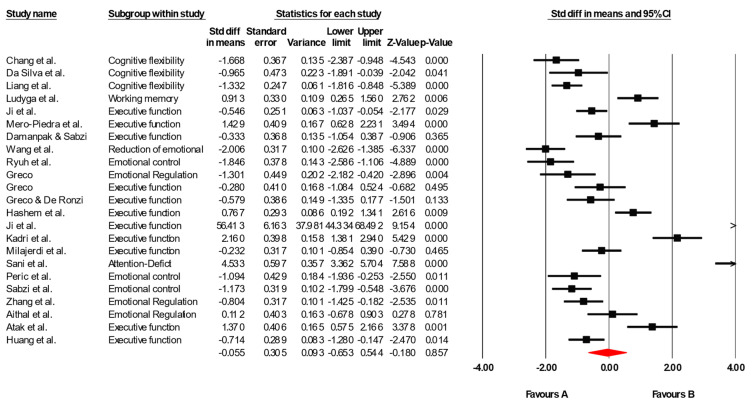
Forest plot showing the effect of physical–motor interventions on executive functioning. Each line represents an individual study with its standardized mean difference (Hedges’ g) and 95% confidence interval (CI). Negative values indicate effects favoring the control group, whereas positive values indicate effects favoring the intervention group. The size of the squares is proportional to the weight of each study. The diamond represents the pooled effect size under a random effects model, with its width corresponding to the 95% CI. The vertical line at zero indicates the line of no effect [26,27,28,29,30,31,32,33,34,35,36,37,38,39,40,41,42,43,44,45,47,48].

A total of 22 studies were included in the meta-analysis to examine the effects of physical–motor interventions on executive functions, cognitive flexibility, working memory, ADHD symptoms, and emotional regulation in children and adolescents with neurodevelopmental disorders. The heterogeneity analysis revealed a Q value of 11.936 with 21 degrees of freedom, suggesting a low between-study heterogeneity. Consistent with this, the I^2^ statistic was 0%, indicating that all variability in effect sizes can be attributed to sampling errors rather than true between-study differences. Furthermore, the Tau-square and Tau values were both 0.000, confirming the absence of dispersion among the estimated true effects. These results strongly support the homogeneity of the reported effect sizes. Given this homogeneity, a fixed effects model was used for the analysis. The overall effect size was −0.0555, with a 95% confidence interval between −0.653 and 0.544. While this average value was not statistically significant (*p* = 0.857), significant and clinically relevant effects were observed in several individual studies, such as those by Chang et al. [26], Ludyga et al. [29], Ryuh et al. [34], and Sani et al. [41], among others. Importantly, negative values indicate an improvement in executive functions and emotional self-regulation in favor of the intervention group. Figure 2 visually represents these results using a forest plot, which shows consistency in the direction of the effects and the limited overlap of the confidence intervals in studies with significant differences.

#### 3.5.1. Subgroup Analysis

A subgroup analysis was performed using the three variables used. A subgroup analysis was also performed on the mean age of participants and another on the total duration of the training sessions. The results revealed significant statistical significance, supported by moderate and inversely negative Hedge’s g effect sizes. Subgroup analyses demonstrated consistent effect sizes across all cases.

##### Executive Function

**Figure 3:**
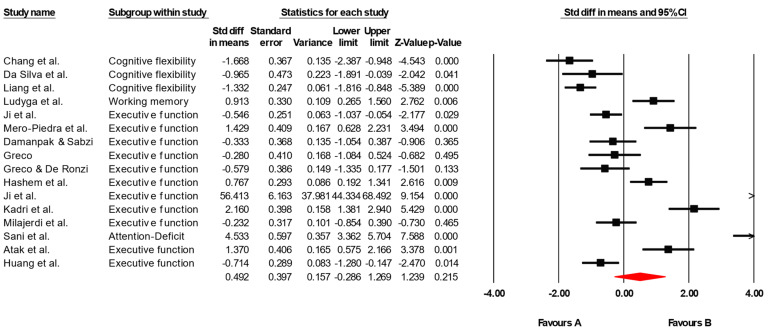
Forest plot displaying subgroup analyses of physical–motor interventions on executive functioning. Hedges’ g with 95% CIs are presented for each subgroup, as well as the overall pooled effect size (diamond). Negative values reflect effects favoring the control group; positive values reflect effects favoring the intervention group. The vertical axis represents the line of no effect (g = 0) [26,27,28,29,30,31,32,35,36,37,38,39,40,41,47,48].

**Figure 4:**
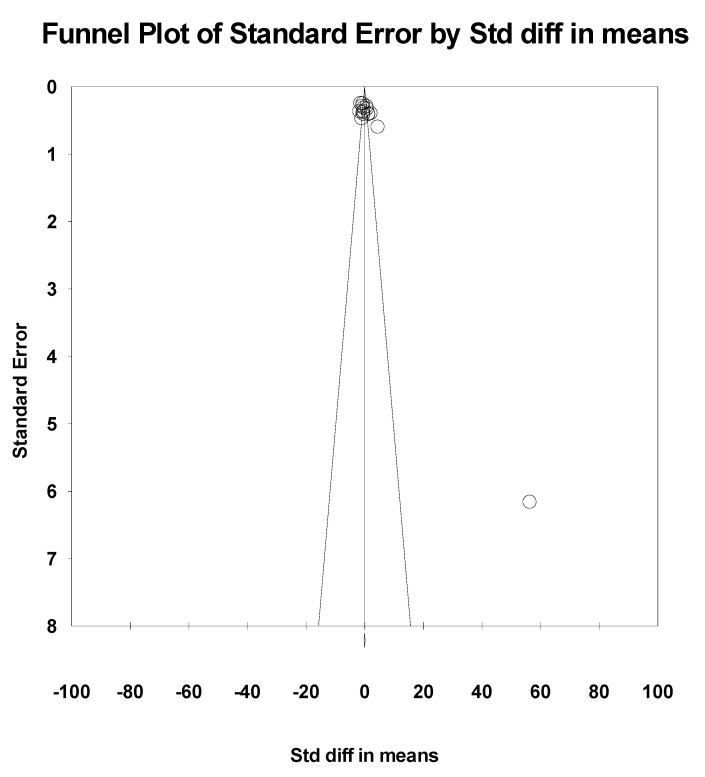
Funnel plot for executive functions.

The results of the meta-analysis revealed an overall standardized effect size (g) of 0.492, indicating a small-to-moderate positive effect of physical interventions on executive functions. However, this effect did not reach statistical significance (*p* = 0.215), as the 95% confidence interval [−0.286, 1.269] includes zero, suggesting uncertainty about the presence of a true effect (Figure 3). To assess between-study heterogeneity, a Q test was conducted, yielding a Q value of 277.579 with 15 degrees of freedom, which is statistically significant (*p* < 0.05), indicating substantial heterogeneity among the included studies. This is further supported by the I^2^ statistic of 94.6%, meaning that approximately 95% of the variability across studies can be attributed to real differences rather than chance. Additionally, the Tau-squared value of 2.227 and Tau of 1.492 confirm a high degree of dispersion in true effect sizes across studies. Therefore, the use of a random effects model was appropriate in this analysis, accounting for variability in effects due to differences in study design, populations, or interventions. Although the Egger’s test (*p* = 0.001) suggests some potential for publication bias, this should be interpreted with caution due to the relatively small number of studies included and the presence of high heterogeneity (Figure 4).

##### Emotional Regulation

**Figure 5:**
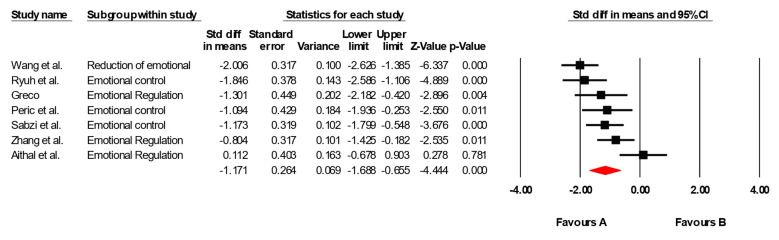
Forest plot showing the effect of physical–motor interventions on emotional regulation. Negative effect sizes indicate improvements in the intervention group relative to the control group, whereas positive values indicate improvements in the control group. Each study is represented by its Hedges’ g and 95% CI, with the diamond denoting the overall pooled effect size under a random effects model [33,34,35,42,43,44,45].

**Figure 6:**
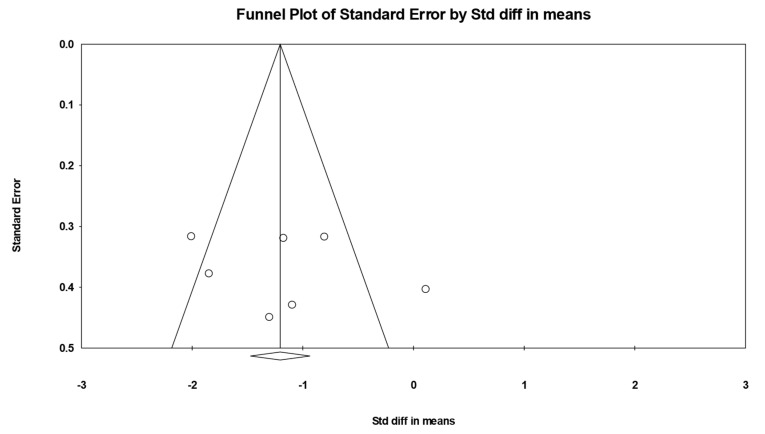
Funnel plot for emotional regulation.

The results of the meta-analysis revealed an overall standardized effect size (g) of −1.204, indicating a large and statistically significant effect of physical interventions on emotional regulation, in favor of the experimental group. This effect was statistically significant (*p* = 0.000), and the 95% confidence interval [−1.688, −0.655] does not include zero, confirming the presence of a reliable and robust effect (Figure 5). To assess between-study heterogeneity, a Q test was conducted, yielding a Q value of 21.667 with 6 degrees of freedom, which is statistically significant (*p* = 0.001). This indicates the presence of substantial heterogeneity across the included studies. The I^2^ statistic was 72.31%, suggesting that a large portion of the variability in effect sizes is due to actual differences among the studies rather than random error. Moreover, the Tau-squared value (0.347) and Tau (0.589) reflect a moderate-to-high dispersion in the true effect sizes, further justifying the application of a random effects model in the analysis. Lastly, Egger’s test revealed potential publication bias (*p* = 0.029), suggesting some asymmetry in the distribution of effect sizes. However, this should be interpreted with caution due to the small number of studies included and the existing heterogeneity (Figure 6).

##### Groups of Age

In the subgroup analysis (CMA, random effects) with children aged 5–11 years old as the reference and adolescents aged 12–17 years old as the secondary level (k = 5), no significant differences were observed between subgroups (Q_between = 1.25, df = 1, *p* = 0.264).

The estimated pooled effect for children aged 5–11 years old was g = 3.93 (95% CI 0.03 to 7.82, *p* = 0.048). The contrast for adolescents aged 12–17 years old versus boys was Δg = −3.21 (95% CI −8.85 to 2.43, *p* = 0.264), implying an effect size of approximately g ≈ 0.72 in adolescents, although with considerable uncertainty. Heterogeneity remained very high (I^2^ ≈ 97.8%); therefore, these results should be interpreted with caution given the scarcity of studies by age group.

##### Total Training Duration

When stratified by total dose into three categories (<720 min, 720–1440 min, and >1440 min), the between-subgroup contrast was Q_between = 5.05, df = 2, *p* = 0.080 (random effects model in CMA). Taking 720–1440 min as the reference, the estimated effect for that subgroup was g ≈ 0.41 (*p* = 0.366); the <720 min subgroup did not differ from the reference (β = +0.16, *p* = 0.848), whereas the >1440 min subgroup showed a significantly smaller effect (β = −1.37, *p* = 0.043). Overall, the pattern suggests that a moderate dose (720–1440 min) tends to be associated with more favorable effects, whereas prolonged exposures (>1440 min) do not provide additional benefits and may even attenuate them (direction based on effect size coding). These findings should be interpreted with caution, given the unequal number of studies per category and the residual heterogeneity.

## 4. Discussion

The primary objective of this study was to synthesize and quantify the available scientific evidence on the effects of physical activity-based interventions on executive functions and emotional regulation in children and adolescents with neurodevelopmental disorders (NDDs), such as ADHD, autism spectrum disorder, intellectual disability, and developmental coordination disorder. Through a meta-analysis of 22 randomized clinical trials, the magnitude of the impact of different modalities of structured physical exercise on key cognitive and emotional variables was assessed, with the aim of guiding future intervention and research.

The results obtained show that physical–motor interventions have a positive, albeit heterogeneous, effect on executive functions and a notable influence on emotional regulation. Specifically, the overall analysis of executive functions revealed a standardized effect size of g = 0.492; however, this result did not reach statistical significance (*p* = 0.215), and the confidence interval included zero (95% CI: −0.286 to 1.269), underscoring the uncertainty regarding the presence of a true effect. Therefore, although the point estimate lies within the small-to-moderate range, these findings should be interpreted with caution and cannot be considered conclusive. In contrast, the meta-analysis of emotional regulation showed an effect size of g = −1.204, which was statistically significant (*p* < 0.001) and clinically relevant, with a 95% CI of [−1.688, −0.655], indicating a robust improvement in emotional regulation in favor of the experimental group. The high levels of heterogeneity observed in both variables (I^2^ = 94.6% for executive functions; I^2^ = 72.3% for emotional regulation) support the use of random effects models, suggesting that the variability between studies is not attributable solely to sampling error but also to real differences in intervention designs, implementation contexts, or participant characteristics.

Regarding methodological quality, most of the included studies had a moderate score on the PEDro scale, with values ranging from 4 to 7 points out of 10. Although all trials were randomized and most reported group comparability at baseline and key outcome measures, a systematic lack was observed in aspects such as the blinding of participants, therapists, and assessors, which could introduce performance and detection bias. Only a small number of studies met the criteria for allocation concealment or intention-to-treat analysis, which limits the robustness of the evidence. This pattern of methodological quality is consistent with that reported in previous reviews of non-pharmacological interventions in populations with neurodevelopmental disorders [48], where the nature of the physical interventions makes complete blinding difficult. However, it is worth noting that studies with higher methodological quality tended to report more consistent and detailed effects, reinforcing the need for standardized design and transparency in future research to strengthen the internal and external validity of the findings.

The included studies were conducted in diverse cultural and educational contexts, such as China, Brazil, Iran, and Italy, which likely influenced the effectiveness and acceptability of physical activity interventions. Recent research highlights that the school context is a key factor in the success of physical activity interventions as institutional goals, teacher support, school climate, and available resources influence both implementation and outcomes. For example, an ethnographic study in primary schools in the United Kingdom showed that differences between schools in organizational culture and educational support determined the effectiveness of physical activity programs [49]. In addition, studies conducted in China have shown that cultural and pedagogical adaptation is crucial for increasing participation levels, whereby the use of the Behavior Change Wheel made it possible to design an intervention tailored to the Chinese school framework [50], while research based on the YPAP model confirmed the mediating role of parental support and physical education in student participation [51]. Other studies also indicate that the perception of barriers and the school climate significantly mediate the practice of physical activity in adolescents [52]. Likewise, it has been proposed to rethink the design of school interventions from a contextualized perspective, which takes into account both the opportunities and the specific limitations of each environment [53,54]. These findings suggest that the effects of physical and motor interventions observed in our review cannot be interpreted outside the cultural and educational systems in which they are implemented, reinforcing the need for future trials to report, in detail, the contextual characteristics of the school and sociocultural environments where they are implemented.

Executive functions are higher-level cognitive processes that include working memory, response inhibition, planning, sustained attention, and cognitive flexibility [55]. These skills are critical for behavioral control, emotional self-regulation, and academic learning, and are often particularly compromised in children and adolescents with NDDs, such as ADHD, ASD, and intellectual disability [56]. In this review, several studies demonstrated significant improvements in executive functions following structured physical exercise programs. For example, Chang et al. [26] and Ludyga et al. [29] reported positive effects on cognitive flexibility and inhibitory control following table tennis and judo interventions, respectively. Liang et al. [28] and Ji et al. [30] also demonstrated improvements in working memory and inhibition through combined programs and stationary bicycle exercise. These positive effects are observed even in clinical or school settings, suggesting relevant practical applicability. These findings are aligned with research that highlights the impact of physical exercise on the activation and plasticity of the frontoparietal networks involved in executive control [57,58]. At the neurobiological level, regular exercise has been shown to improve the functional connectivity and volume of regions such as the dorsolateral prefrontal cortex and striatum, which are essential for executive processing [59].

Furthermore, systematic reviews and meta-analyses in adults reinforce this evidence. For example, Ludyga et al. [60] and Ham et al. [61] reported consistent improvements in executive functions after aerobic and strength training programs in older adults, including those with mild cognitive impairment. In a more recent review, Xue et al. [62] confirmed that multicomponent exercise interventions (combining aerobic, strength, and coordination exercises) are particularly effective in enhancing working memory and cognitive flexibility, both in young people and adults. The high heterogeneity observed across studies (I^2^ = 94.6%) indicates that the effects of physical–motor interventions are not uniform and may be modulated by various factors. Our subgroup analyses showed that both participant age (children vs. adolescents) and total training dose (<720, 720–1440, and >1440 min) could contribute to explaining part of this variability, although the limited number of studies in each subgroup restricted the statistical power of the results. Beyond these moderators, other variables such as the specific type of neurodevelopmental disorder, exercise intensity, or activity modality (structured vs. unstructured exercises; individual vs. group activities) are also plausible. In this sense, interventions with a clear structure and defined rules—such as martial arts, team sports, or organized motor games—appear especially promising for improving inhibitory control, decision-making, and strategic planning [63]. However, the available evidence is still insufficient to confirm these hypotheses, reinforcing the need for future trials to systematically report on intervention intensity and comparatively explore different formats of physical activity. In contrast, brief or low-intensity interventions, such as a single 20–30 min session, tend to show inconsistent effects, as reflected in the study by Mero Piedra et al. [31]. Furthermore, the potential risk of publication bias must be considered, as suggested by the result of the Egger test (*p* = 0.001), which indicates that studies with positive results may be overrepresented in the literature. This tendency could overestimate the true effect of the interventions. Therefore, further research is needed with robust designs, large samples, longitudinal follow-up, and the use of standardized measures of executive function that allow comparability across studies. It would also be relevant to evaluate the possible mediating mechanisms, such as motivation, social engagement, or emotional regulation, which could amplify or moderate the effect of exercise on executive functions.

Emotional regulation is an essential component of psychological functioning, especially in children and adolescents with NDDs, who often have difficulty identifying, modulating, and expressing their emotions adaptively [64]. Disorders such as ADHD, ASD, and intellectual disability are frequently associated with emotional dysregulation, impulsivity, anxiety, and internalizing and externalizing behavior problems [7]. In this systematic review, several studies showed significant improvements in emotional variables after structured physical exercise interventions. For example, Wang et al. [33] observed a significant reduction in emotional and behavioral problems after a sensory integration-based intervention. Ryuh et al. [34] reported improvements in emotional control in children with ADHD after participating in a playful educational program. Similarly, Greco [35] and Greco and De Ronzi [36] found that multicomponent programs, including physical activity and social games, improved both emotional self-regulation and adaptive behavior in children with ASD. These results are consistent with the scientific literature linking physical exercise with the positive modulation of the hypothalamic–pituitary–adrenal (HPA) axis, increased levels of neurotransmitters such as serotonin and dopamine, and greater functional connectivity in limbic networks responsible for emotional processing [65,66]. In adults, meta-analyses such as that by Schuch et al. [67] have shown that regular exercise can be as effective as pharmacological treatments in reducing mild and moderate depressive symptoms, also suggesting relevant transdiagnostic effects in young people with NDDs. A recent review by Pascoe et al. [68] on exercise and psychological stress in adolescents showed that physical activity acts as a buffer against chronic stress, reducing anxiety symptoms and improving emotional resilience. Similarly, Biddle et al. [69] found that interventions based on active play and aerobic exercise improve mood and reduce anxiety symptoms in school-aged children. Despite these promising findings, considerable methodological heterogeneity (I^2^ = 72.3%) was also detected, both in the measures used to assess emotional regulation (CBCL, SDQ, and CPRS-R, among others) and in the duration, modality, and context of the interventions. Our subgroup analyses suggest that the total training dose and the age of the participants could contribute to explaining some of this variability, although the small number of studies in each subgroup limited the statistical power of the results. Other factors that could be an influence include the type of neurodevelopmental disorder, the intensity of the sessions, and the format of the activity (structured vs. free; individual vs. group). Interventions with a clear structure and defined rules seem to be associated with more consistent effects, while brief or unstructured programs tend to yield mixed results. Furthermore, some studies, such as those by Perić et al. [42] and Sabzi et al. [43], reported improvements in reducing aggression, anxiety, and depressive symptoms in young people with intellectual disabilities or ADHD following aquatic or motor programs, highlighting the importance of the therapeutic environment and accessibility. Overall, although the effects of physical exercise on emotional regulation in children and adolescents with ODD are encouraging, the literature still presents important gaps. More randomized clinical trials of high methodological quality are needed to explore specific mechanisms of action, consider contextual variables (family, school, and socioeconomic status), and analyze the sustainability of long-term effects. Furthermore, it would be advisable for future studies to systematically report exercise intensity and use psychophysiological measures (cortisol, heart rate, and functional resonance) to objectively triangulate the reported emotional outcomes. Despite the promising findings, this systematic review and meta-analysis presents several limitations that should be considered when interpreting the results. First, substantial methodological heterogeneity was observed across the included trials, both in the characteristics of the physical–motor interventions (e.g., type, duration, frequency, and intensity) and in the outcome measures employed. Executive functions were mostly assessed using standardized neuropsychological tests, whereas emotional regulation was frequently measured through parent- or teacher-reported questionnaires (e.g., CBCL, SDQ, and CPRS-R), which may have reduced objectivity and contributed to inconsistency. Second, the overall methodological quality of the studies was moderate according to the PEDro scale, with poor compliance in domains such as the blinding of participants and assessors or the use of intention-to-treat analyses, which can introduce systematic bias. Third, many of the included RCTs relied on small sample sizes, which not only limits statistical power but may also inflate effect size estimates. Fourth, potential publication bias must be considered, as suggested by the Egger test for some outcomes, whereby studies with null or negative findings are likely underrepresented in the literature, which may have led to an overestimation of positive effects and limits the generalizability of results to clinical practice. Additionally, heterogeneity in diagnostic frameworks (DSM-IV, DSM-5, and ICD-10) may have reduced comparability between studies. Additionally, the analysis combined different neurodevelopmental disorders (ADHD, ASD, ID, and DCD) into a single group, limiting disorder-specific interpretability. Finally, exercise intensity was not consistently reported (e.g., %HRmax and RPE), precluding further moderator analyses according to this variable. Future trials should therefore (i) increase methodological rigor, (ii) recruit larger and more diverse samples, (iii) systematically register and publish results regardless of outcome, (iv) adopt standardized diagnostic frameworks, and (v) provide detailed reports of exercise intensity and use multimodal, objective measures of emotional regulation. These improvements would allow for more robust comparisons and enhance the applicability of the findings to clinical and educational practice. 

## 5. Conclusions

The results of this systematic review and meta-analysis suggest that physical–motor interventions have an overall positive impact on executive functions and emotional regulation in children and adolescents with neurodevelopmental disorders (NDDs), especially in populations with ADHD, ASD, intellectual disability, and developmental coordination disorder. Although effect sizes vary depending on the function assessed and the characteristics of the intervention, most of the included studies show significant improvements in domains such as working memory, cognitive flexibility, sustained attention, and inhibitory control, as well as in variables related to emotional well-being, behavior, and social skills. Evidence supports the usefulness of structured physical activity, especially those modalities that involve coordination, planning, and emotional regulation, such as combat sports, combined exercise, or play-based programs, as a complementary therapeutic tool in addressing cognitive and emotional difficulties in this population. However, due to the heterogeneity of the included studies, the variability in the assessment tools, and certain methodological limitations, it is recommended that the results be interpreted with caution. It is necessary to continue developing research with greater experimental rigor, longitudinal follow-up, and direct comparisons between intervention types to optimize the prescription of physical exercise in educational, clinical, and community settings.

## Figures and Tables

**Table 1:** Methodological quality of the included articles.

Study	1	2	3	4	5	6	7	8	9	10	11	Total Score
Chang et al. [26]	1	1	0	1	0	0	0	1	0	1	1	5/10
Da Silva et al. [27]	1	1	0	1	0	0	0	0	0	1	1	4/10
Liang et al. [28]	1	1	0	1	0	0	0	1	0	1	1	5/10
Ludyga et al. [29]	1	1	1	0	0	1	1	1	0	1	1	7/10
Ji et al. [30]	1	1	0	1	0	0	0	0	0	1	1	4/10
Mero Piedra et al. [31]	1	1	0	0	0	0	0	1	0	1	1	4/10
Damanpak & Sabzi [32]	1	1	0	0	0	0	1	1	0	1	1	5/10
Wang et al. [33]	1	1	0	0	0	0	1	1	0	1	1	5/10
Ryuh et al. [34]	1	0	0	0	0	1	1	1	0	1	1	5/10
Greco [35]	1	1	0	0	0	0	1	1	0	1	1	5/10
Greco & De Ronzi [36]	1	1	0	0	0	0	1	1	0	1	1	5/10
Hashemi et al. [37]	1	1	0	0	0	0	1	0	0	1	0	5/10
Ji et al. [38]	1	1	0	0	0	1	1	1	0	1	1	6/10
Kadri et al. [39]	1	1	0	1	0	0	0	0	0	1	1	4/10
Milajerdi et al. [40]	1	1	0	1	0	0	0	1	1	1	1	6/10
Sani et al. [41]	0	1	0	1	0	0	1	0	0	1	1	5/10
Perić et al. [42]	1	1	0	1	0	0	0	1	0	1	1	5/10
Sabzi et al. [43]	1	1	0	1	0	0	0	1	0	1	1	6/10
Zhang et al. [44]	1	1	0	1	0	0	0	1	1	1	1	6/10
Aithal et al. [45]	1	1	0	1	0	0	0	1	0	1	1	5/10
Atak et al. [46]	0	1	0	1	0	0	1	1	0	1	1	6/10
Huang et al. [47]	1	1	0	1	0	0	0	1	0	1	1	5/10

Items: 1: eligibility criteria; 2: random allocation; 3: concealed allocation; 4: baseline comparability; 5: blind subjects; 6: blind therapists; 7: blind assessors; 8: adequate follow-up; 9: intention-to-treat analysis; 10: between-group comparisons; 11: point estimates and variability; yes = 1; no = 0.

**Table 2:** Characteristics of the included studies.

**Authors**	Study Design/ Setting	Type of NDD; Diagnosed Methods	Age	Sample size (Exercise/ Control)	Type of Physical Activity	Session Duration, Frequency	Total Sessions, Total Duration	Outcomes and Measures	Results
Chang et al. [26]	RCT/School	ADHD; DSM-5	8.3 ± 1.3	40 (20/20)	Table tennis (CEE)	60 min, 3x/week	36 sessions, 2160 min	WCST, Stroop Word and Color Test	Cognitive flexibility and inhibitory control improved (*p* = 0.002 and *p* = 0.017)
Da Silva et al. [27]	RCT/University	ADHD; DSM-IV	12.1 ± 1.6	20 (10/10)	Swimming	45 min, 2x/week	16 sessions, 720 min	Test of trails	Cognitive flexibility (*p* = 0.042)
Liang et al. [28]	RCT/Not reported	ADHD; DSM-5	8.5 ± 1.5	80 (40/40)	Combined exercise	60 min, 3x/week	36 sessions, 2160 min	Flanker, Tower of London, Trail Making Test	Improvements in working memory and cognitive flexibility (*p* < 0.01)
Ludyga et al. [29]	RCT/Not reported	ADHD; DSM-5	10.4 ± 13	41 (23/18)	Judo (CEE)	60 min, 2x/week	24 sessions, 1440 min	Change Detection paradigm	Working memory improved (K-score *p* = 0.030)
Ji et al. [30]	RCT/Not reported	ASD; ICD-10	12.8 ± 2.7	66 (33/33)	Physical exercise	60 min, 3x/week	27 sessions, 1620 min	Digit span, Flanker test, Stroop test	Improvements in memory and inhibition (*p* < 0.001)
Mero Piedra et al. [31]	RCT/Not reported	Intellectual disability; Clinically diagnosed	12.7 ± 1.35	30 (15/15)	Physical education	60 min, 2x/week	12 sessions, 720 min	Executive function, Inhibition, Interference	No significant differences (*p* = 0.94, *p* = 0.13)
Damanpak & Sabzi [32]	RCT/Not reported	DCD; DCDQ	10.7 ± 0.5	30 (15/15)	Motor games	60 min, 3x/week	24 sessions, 1440 min	Coolidge Executive Functioning Scale	Improvements in planning, organization, and inhibition (*p* = 0.001)
Wang et al. [33]	RCT/Not reported	ASD; DSM-5	7 ± 0.8	60 (30/30)	Sensory integration	40 min, 2x/week	16 sessions, 640 min	CBCL, PSQ, SSRS	Reduction in emotional and behavioral problems (*p* < 0.001)
Ryuh et al. [34]	RCT/Clinic	ADHD; DSM-5	10.8 ± 0.7	40 (20/20)	Educational games	60 min, 2x/week	16 sessions, 960 min	Stroop test, Emotional control scale	Improvements in emotional control and inhibitory control
Greco [35]	RCT/Clinical	ASD; ADOS-2	9.3 ± 0.92	24 (12/12)	Not reported	70 min, 2x/week	24 sessions, 1680 min	Behavior Rating Inventory of Executive Function	Improvements in emotional, cognitive, and behavioral regulation (*p* < 0.05)
Greco & De Ronzi [36]	RCT/Clinical	ASD; ADOS-2	9.1 ± 1.0	28 (14/14)	Karate training	45 min, 2x/week	24 sessions, 1080 min	EF: BRIEF	Significant improvements in executive functions
Hashemi et al. [37]	RCT/School	DCD; DSM-V	9.6 ± 2.24	50 (25/25)	Usual care	30 min, 3x/week	24 sessions, 720 min	EF: CAS-planning, Attention: CAS-attention, Memory: TVPS-R	Improvements in attention, memory, and planning
Ji et al. [38]	RCT/Clinical	ADHD; Clinical diagnosis	9.0 ± 1.5	42 (21/21)	Stationary bike exercise	50 min, 3x/week	12 sessions, 600 min	EF: GNG; Attention: FAIR	Improvements in sustained attention and inhibitory functions
Kadri et al. [39]	RCT/School	ADHD; Psychologist	14.4 ± 3.22	40 (20/20)	Taekwondo Practice	50 min, 2x/week	Approx. 144 sessions	EF: Stroop Task; Attention: Ruff 2 and 7	Improvements in selective attention and inhibitory control
Milajerdi et al. [40]	RCT/School	ASD; ADOS-2	8.2 ± 1.5	40 (20/20)	Exergaming	35 min, 3x/week	24 sessions, 840 min	EF: WCST	Improvements in cognitive flexibility
Sani et al. [41]	RCT/Clinical	ADHD; DSM-V	7.5 ± 1.3	25/25	Neurofeedback	≈42 min, 3x/week	20 sessions, ~840 min	Attention: CPT	Significant improvements in sustained care
Perić et al. [42]	RCT	ID; WISC	15.7 ± 0.5	25 (12/13)	No PA	60 min, 2x/week	32 sessions, 1920 min	Psychosocial variables assessment	Significant reduction in aggression, anxiety, and depression (*p* < 0.05)
Sabzi et al. [43]	RCT/ NR	ADHD; CPRS-R	9.5 ± 0.5	46(23/23)	Water treadmill	30 min/session, 3 times/week	24 sessions, 720 min	Conduct problems, Anxiety: Conner’s Parent Rating Scale—Revised	Externalizing problems Conduct problems (*p* = 0.003) Internalizing problems Anxiety (*p* = 0.017)
Zhang et al. [44]	RCT/ Clinical	ADHD; DSM-5	8.8 ± 1.42	43(22/21)	Motor skills training	60 min/session, 3 times/week	36 sessions, 2160 min	Quality of life: Pediatric Quality of Life Inventory	Psychological well-being Quality of life (*p* = 0.046)
Aithal et al. [45]	RCT/ NR	ASD; DSM-5	10.7 ± 1.25	26(10/16)	Dance movement	40 min/session, twice/week	10 sessions, 400 min	Emotional and social well-being: Strengths and Difficulties Questionnaire	Psychological well-being Emotional and social well-being (*p* = 0.02)
Atak et al. [46]	RCT/ NR	ID; WISC-R	8.7 ± 1.6	30(15/15)	Balance training	30 min/session, twice/week	24 sessions, 720 min	Attention, impulsivity: MOXO attention scale	Cognitive function Attention (*p* = 0.001) Externalizing problems Impulsivity (*p* = 0.003)
Huang et al. [47]	RCT/ School	LD; DSM-4	12 ± 0.81	51(25/26)	Acute aerobic exercise	30 min/session	1 session, 30 min	Sustained attention: sustained attention test	Cognitive function Sustained attention (*p* < 0.05)

MVPA: moderate-to-vigorous physical activity; ADOS-2: Autism Diagnostic Observation Schedule, 2nd edition; DSM-5: Diagnostic and Statistical Manual of Mental Disorders, 5th edition; FAIR: Frankfurt Attention Inventory; EF: executive functions; BRIEF: Behavior Rating Inventory of Executive Function; CBCL: Child Behavior Checklist; CPT: Continuous Performance Test; CAS: Cognitive Assessment System; ADHD: attention-deficit/hyperactivity disorder; ICD-10: International Classification of Diseases, 10th revision; SSRS: Social Skills Rating System; DCDQ: Developmental Coordination Disorder Questionnaire; GNG: Go/No-Go Task; TVPS-R: Test of Visual Perceptual Skills—Revised; WISC: Wechsler Intelligence Scale for Children; RCT: randomized controlled trial; ASD: autism spectrum disorder; CEE: controlled exercise environment; WCST: Wisconsin Card Sorting Test; ID: intellectual disability; DSM-IV: Diagnostic and Statistical Manual of Mental Disorders, 4th edition; DCD: developmental coordination disorder; PSQ: Pediatric Symptom Checklist.

## Data Availability

No new data were created or analyzed in this study.

## Abbreviations

The following abbreviations are used in this manuscript:

NDD	Neurodevelopmental disorder
ADHD	Attention-deficit/hyperactivity disorder
ASD	Autism spectrum disorder
ID	Intellectual disability
DCD	Developmental coordination disorder
PA	Physical Activity
RCTs	Randomized clinical trials
WOS	Web of Science
HPA	Hypothalamic–pituitary–adrenal
